# The Talent Development Pathway for Elite Basketball Players in China

**DOI:** 10.3390/ijerph17145110

**Published:** 2020-07-15

**Authors:** José Bonal, Sergio Lorenzo Jiménez, Alberto Lorenzo

**Affiliations:** 1 Sport Department, Faculty of Sport Sciences, Universidad Europea de Madrid, 28670 Madrid, Spain; 2 Sport Department, Faculty of Sport Sciences, Universidad Politécnica de Madrid, 28040 Madrid, Spain; alberto.lorenzo@upm.es

**Keywords:** talent development, basketball, China, cultural factors, qualitative research

## Abstract

A large portion of previous sport talent development research has been conducted using Western countries study subjects such as Canadian, Swedish, Spanish, British, or American athletes. However, the factors that affect oriental culture athletes remain an unexplored field. The aims of this investigation were to consolidate the exploration of the pilot study that studied the key factors for Chinese elite basketball players’ careers and understand what facts have helped them to achieve the highest sportive level through qualitative research. The pathway to excellence of 11 Chinese elite basketball players were analyzed through a semi-structured interview with different categories such as social context, sport context, tactical factors, or anthropometric factors. Results showed that cultural factors, family tradition, academic studies, coaches, mental strength, training structuration, and international competitions had a great effect and influence in the talent development of Chinese basketball players.

## 1. Introduction

China, which has sustained a great socio-economic evolution in recent decades [1], has become a country with great sports potential. This is endorsed by the 546 medals that it treasures in its Olympic history, with which they will reach the Tokyo 2021 Summer Olympics as the seventh highest medal earning country (despite only having participated 10 times, beginning in 1952 followed by an uninterrupted period of 1984–2016). Contrary to what is observed in this sports performance trend, in one of the most practiced sports worldwide, basketball, the results of China are not as successful. In their history, they have achieved three Olympic medals (two silver and a bronze in total, all in the women’s category) and two podiums in world championships (one silver and one bronze, also in the women’s category). As part of the performance plan built to maximize sportive results at the Olympic Games in Beijing 2008, a pyramidal talent development and talent identification system was created and implemented in all of the Chinese provinces [2]. This program is composed by four levels and focuses on the use of battery tests for early talent detection (reaching children up to the age of six), aiming to recruit the greatest natural talents [3,4,5]. Existing literature on this approach is limited [6,7] due to the lack of replicability in comparison to the real competition situations, e.g., fatigue and other external conditions are not contemplated [8,9,10]. This kind of studies that consider talent as a fixed capacity, without evolution through the time, and that need to be identified early, have been classified as weak (e.g., references [11,12]) supported by the idea that early performance indicators (e.g., height) have very little connection with the determinants of success at the adult level (i.e., the groups are mainly homogeneous in these characteristics) [7]. Thus, as many authors stated the idea that many of players have talent, but it is more important to be able to develop it [13,14,15,16,17,18], so it is necessary to value more the individual path followed by each athlete and to value more the precise moments to develop training processes appropriate to each age [9]. To date, there are some studies that investigate not only talent detection but the context of athletes’ talent development in China [19] and Hong Kong [20,21].

There are multiple factors that intervene in athlete talent development such as genetics, training structure, coach influence, family influence, psychological traits, etc. [22]. These factors have been studied with different approaches, focusing on athlete talent development career transitions [18,23,24,25] or talent development as a holistic phenomenon [13,14,16,26,27]. In both cases, a vital itinerary has been a recurrent tool to study in depth elite career pathways [8,23,28,29,30,31,32,33,34], since it allows the investigators to understand the factors affecting the athlete and his circumstances. Stambulova et al. [24] already talked about the importance of such circumstances, especially when considering their cultural framework. Regarding this matter, there is important to highlight that most of vital itinerary studies have been carried out using predominantly western culture multi-sport sample (e.g., British Olympic athletes [31], American gymnasts [28], Swedish tennis players [29], or European footballers [30]) or specific basketball field studies (elite Spanish basketball players [35,36], Brazilian female world champions [37], or Portuguese male [38] and female players [39]). Considering this, there seems to be a knowledge gap about this phenomenon in the case of East Asian countries. To fill this gap, the present study is proposed, whose objective is to determine and analyze in depth the factors that affect the development of elite basketball talent in China.

## 2. Materials and Methods

### 2.1. Design

The present investigation is part of a larger research project with professional Chinese basketball players (after a preliminary pilot study [40]) that aims to explore the key factors for talent development that influence Chinese basketballers in all different dimensions. Qualitative methodology and semi-structured interview as the research instruments were chosen [41].

### 2.2. Participants

Eleven Chinese high-level players were included in the present study. To ensure the highest research quality, the “ideal typical case” [1] was applied as the sample selection criteria.

The inclusion criteria were chosen accordingly to the definition characteristics described by Swann et al. [42,43], affirming that the sample used at the present investigation qualifies as “world class elite”—the highest level of elitism rank. In our case, all subjects (a) had more than 10 years of experience at the highest level of competition, (b) have played international championships with their respective teams or with the Chinese national team (Olympic Games, World Championships, or Asian Championships), and (c) have participated in the final phase of their leagues and they achieved some titles in the competitions they played. Besides all of this, we must consider that basketball is one of the most popular sports of China, with a greater number of clubs and participants and a competitive structure.

Undoubtedly, this is one of the strengths of this study, since to date the most of studies with high scientific impact on the development of talent in basketball in China used subjects from youth categories (e.g., Ma [44]) or university players (e.g., Zheng and Yang [45]). It should be noted that the entire protocol of action of this study received the approval of the Ethical Committee of the European University of Madrid with file number CIPI/20/131.

### 2.3. Procedure

The main research tool used in the present investigation was a semi-structured interview that focused on the vital itinerary of the athletes [41]. Eleven high-level Chinese players were interviewed, and the interview consisted of open-ended questions meant to be answered in the form of a monologue in order to further deepen with clarifying questions [46]. This procedure allows the investigators to collect detailed and real information for multiple cases and explore in-depth aspects that may arise during the interview, considering that what it is important for a specific subject may not be important for another. For those reasons, the semi-structured interview is the ideal research approach [47]. The interview script was structured following the proposal of García Martín, Antúnez, and Ibañez [48], described in Table 1.

Additionally some questions were added following the protocol used by Jiménez y Lorenzo [2], including pilot interviews and the interview script reviewed by four PhD qualitative research experts (two Spanish nationals and two fully bilingual Chinese nationals). The average interview time was 39 min, and the used languages were English and Chinese (interpreter assistance was required in 7/11 interviews).

### 2.4. Data Analysis

For the qualitative data analysis, the specific coding software QDA Milner 5.0 was used, which allowed the investigators to follow an inductive approach [49,50] when analyzing the collected information into different “units of meaning” [51]. Those units of meaning were selected by a grammatical criterion (sentence) and the thematical criterion (idea by itself), been revised and grouped into properties, and later, categories. Part of the implications of an inductive methodology include the possibility of, based on interviewees’ answers, the emergence of new properties and categories and new and valuable information with respect novel topics not contemplated by the previous bibliography. To capture the ideas of the interviewees in a precise and effective way, the interview transcription process followed the advice given by Silverman [52] and Gibbs [53], which include aspects such as the immediate transcription of the different interviews when finished and the use of a field diary to capture the subjects’ expressions and impressions.

To facilitate the interviewees with opportunities to modify, add or delete the information provided is an effective way to improve research credibility [54,55,56,57]. In this sense, on the present investigation that circumstance was given on up to two occasions: just after the interview, when asking the interviewee if he would like to modify something, and after literally transcribing the interview carried out by sending a copy of said transcript to the player. Of the 11 subjects, eight did not change anything, one modified two units of meaning, and two did not respond.

A very exhaustive coding process performed by two independent external coders (familiar with qualitative methodology) was followed in order to guarantee the research internal reliability. (a) We first trained the coders [57]; (b) second, the units of meaning were located within each property and category; (c) subsequently, the analysis book was coded without seeing the coding made by the other researcher; (d) after each round of analysis, the agreement between coders was assessed, disagreements were reviewed and coded again until reaching a value of 93.4%; and (e) intercoder agreement was measured following the proposal by Miles and Huberman [56], where intercoder reliability is equal to the number of agreements divided by the number of total agreements plus disagreements.

## 3. Results

The study results were analyzed quantitatively and qualitatively.

### 3.1. Results: Quantitative Data Analysis

**Figure 1:**
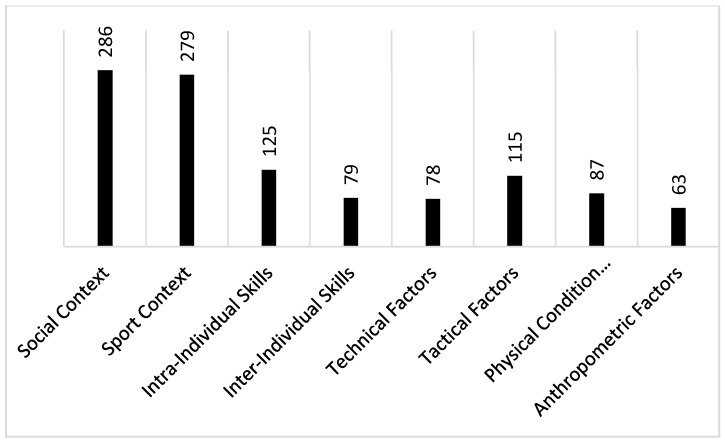
Quantitative data analysis: meaning units coded in the present study.

As Figure 1 shows, a total of 1112 units of meaning (MU) distributed in eight categories and 42 sub-categories or properties were coded as follows:

- “Social Context” (286 US) appeared as the category with highest number of MU.
- “Sports Context” (279 MU).
- “Intra-Individual Skills” (125 MU).
- “Tactical Factors “(115 MU).
- “Physical Capacities” (87 MU).
- “Inter-Individual Skills” (79 MU).
- “Technical Factors” (78 MU).
- “Anthropometric Factors” (63 MU) with the lowest number of MU.

### 3.2. Results: Qualitative Data Analysis

From a qualitative point of view, based on the nature of the results, four groups of properties and categories were grouped into the following four different dimensions:

- The context dimension: made up of the social context category;
- The sports context dimension: made up of the sports context category;
- The psychological factors dimension: product of the union of the categories “intra-individual skills” and “inter-individual skills”;
- The sport specific factors dimension: product of the union of the categories “tactical factors,” “technical factors,” “physical capabilities,” and “anthropometric factors.”

It is also important to highlight the presence of different factors associated with a cultural nature, which intrinsically impregnated each of the other dimensions, which had a remarkable specific weight in the overall research.

**Figure 2:**
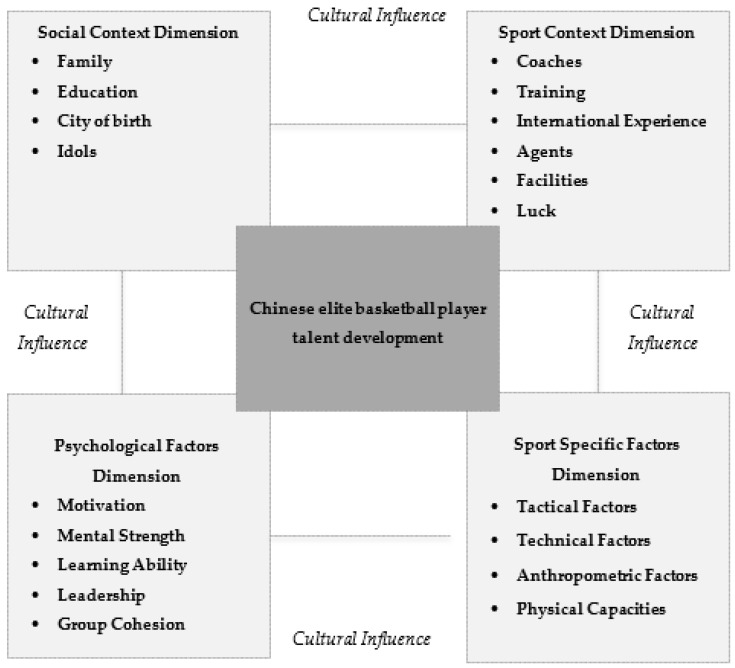
Qualitative data analysis: dimensions affecting talent development of elite basketball players in China.

Figure 2 summarizes all the resulting dimensions and factors around the development of talent of the Chinese elite basketball player and the most important aspects in each of them.

#### 3.2.1. Social Context Dimension

Social context was the most frequent category in the codification of the entire study, both globally and in each of the cases individually. One of the first factors highlighted by absolutely all players was the role played by their family and its positive effect throughout their career in relation to their talent development, supporting them on all different types of challenges (S1, S2, S3, S4, S5, S6, S7, S8, S9, S10, S11).

“My parents were always there, they are the ones who know me the most and the truth was that they were always by my side. It could be said that I have overcome all difficulties in large part, thanks to them”

(S9).

In relation to this topic, we should consider that China is a country in which, for many years, national law did not allow families to have more than one child, except for those who paid a tax within the reach of very few households. It has meant that many of the subjects make a particular and proper mention of Chinese culture within the family factor, with the possible degree of influence of this particularity on their trajectories. (S1, S2, S3, S4, S7, S8, S9, S10, S11). Following this line, it is important to understand the cultural importance for most of the subjects of the concept of “family tradition” throughout their vital itinerary. This is mentioned by almost all subjects (S1, S2, S3, S4, S5, S6, S7, S8, S10) as one of the most influential factors either as a main motivation for playing basketball or for choosing basketball as a life-sport. In addition, it coincides with the circumstance that almost all the parents of the subjects had been professional athletes, both in basketball and other sports disciplines (S1, S2, S3, S4, S5, S6, S7, S10).

“My family has always been a great point of support. When I was 8 years old my parents wanted me to play basketball at all costs. Of course I didn’t want to play then, because I really didn’t like it, but even so, my parents believed that I should play, since basketball is our family tradition. They both had played professionally in the past and believed that I should follow in their footsteps and become a professional player as they were”

(S1).

After the first round of interview analysis, it was inductively observed that there were numerous specific references that, in addition to the Family factor, at the same time depended on the specific culture of the Chinese social context, which is why the culture social context factor emerged. This factor encompasses not only the references to the Chinese “family tradition” but also many other cross-sectional references within the framework of the Chinese cultural social context. One of those factors that it affected transversally was the education factor. Specifically, many players talk about the great importance of studying and the associated positive effects that education has had on their development (S1, S2, S4, S6, S8, S9, S10, S11). Other players not only referred to the influence of the academic education factor but also referred to the cultural nuances of the country (S1, S2, S4, S8, S10, S11). In China, it is quite common to drop out of studies at 13–14 years of age if you are selected to play in one of the high-performance teams or centers. This phenomenon was not perceived as something positive by any of the interviewees.

“I consider academic studies important, I see examples in my classmates like Wei Meng or Han Dejun who have made a career in college, and therefore have a better understanding of what the coach says. In my case, in addition to that, academic studies help me to concentrate better, while many of my colleagues who did not have this academic participation of any kind are mentally exhausted during training or slower when understanding new tactical concepts”

(S6).

With a lower grade of importance inside the social context dimension, players identify two more factors that influence their talent—city of birth and idols. In relation to city of birth, it was stated that “medium-size” cities are the best to develop as a sportive leader, since they allow the good players to stood out much easier than in big metropolises, but at the same time, they provide a minimum sport structure in terms of number of clubs, resources, other socioeconomic benefits such as good family potential jobs, etc. In relation to idols, mostly, they play an inspirational role for players in their youth.

#### 3.2.2. Sport Context Dimension

Very close to the frequencies of the social context, the dimension sports context (279 MU) was located in the first level of importance and frequencies of units of meaning.

The totality of the subjects made many and repeated mentions to the importance of the figure of the trainer throughout their life itinerary, where they highlighted their great positive influence on their talent development process (S1, S2, S3, S4, S5, S6, S7, S8, S9, S10, S11). Curiously, for most of the subjects interviewed, the specific figure of their first coach was especially relevant in their careers, these coaches being more a factor to consider in technical-tactical terms but also a mentor or life guide. (S2, S3, S5, S6, S7, S10, S11). In addition to the previous mentor figure, some players highlight the role of coaches as scouts or talent identifiers in their careers, especially in the initial stages (S3, S6, S7, S10, S11).

“My first coach was key for me. He was taller than other children and from the beginning this coach trusted me to play basketball in clubs. He has supported me all this time, from the beginning until now. He has been a professional and personal mentor, because he not only gave me sports opportunities, but I would dare to say that I grew up almost like a father. He taught me the most important things in basketball and in life”

(S6).

With negative nuances, a very mentioned aspect in the interviews was the one related to overtraining or lack of quality training, related by many of the subjects to a poor preparation of Chinese coaches (compared to foreigners) or to a poor training structure (S1, S2, S3, S6, S7, S8, S10, S11). As in the case of the previous category, many of the subjects made a specific mention of the Chinese cultural context, so after the first round of coding, the Sport Context Culture property emerged inductively. This covered cross-sectionally some cultural aspects related to coaches, their way of acting, the type or structure of training or some other issues specifically understood and expressed under the prism of the idiosyncrasy of Chinese culture. Many of the subjects in the sample played for several seasons in countries such as Greece, the United States, or Australia, which is why, in many of their statements, they compare the sports cultural framework of their different experiences (S1, S2, S3, S4, S7, S8, S10).

“When I was young, I trained three times a day, about six or seven hours a day. This is quite common in China. I think it is not good to train 6 hours a day, it is too much. Although we train a lot, we still cannot reach the level of the United States or other European countries because we are very tired, and that impedes our ability to learn. I had a 15-year-old coach who made us train at least seven hours a day, I was exhausted. … In Australia I train less, but more effectively, I prefer Australia’s training methodology. In Australia it is more scientific, and in China it is pure accumulation of quantity. In Australia there is a large base of physical training, for example controlling nutrition and quality of training. However, Chinese teams spend more hours in training, they value the amount of training over quality”

(S3).

To a lesser degree of relationship with the Chinese cultural context, several of the subjects highlighted the importance and positive effect of being able to experience international experiences both at the club level and with the national team (S1, S2, S3, S4, S7, S8, S9, S10).

To a lesser extent, but also especially prominently for some cases (S1), the subjects assessed the importance and positive influence that the figure of the sports manager or agent can bring to achieve better sports opportunities, especially collaborating positively in the construction of bridges between your team of origin in China and other teams abroad (S1, S7, S8, S11).

Another of the variables mentioned to a lesser extent, with the exception of a case where it was given great importance (S7), was the presence of sports facilities to play basketball, especially the existence of these in early stages of training as a generating factor of attraction in potential new players (S2, S7, S9).

“Now there are more possibilities for people to play, and that means that there are more people who want to play. Basketball is now more popular, for example after Yao’s career. He helped make basketball more popular in China and the infrastructure and facilities grew a lot thanks to that”

(S2).

Finally, in this dimension, the subjects refer to the concept of “luck/opportunity” throughout their careers, although they do not always mention it as associated with the same element (S1, S4, S6, S7, S8, S11). Some subjects refer to this luck and focus on the absence of sports injuries (S4, S6), others focused on the luck of being identified as young talents (S3, S4, S11), on the waste of opportunity because it came at an inappropriate time (S7), and others simply to the general course without major setbacks during his sports career (S1, S8).

#### 3.2.3. Psychological Factors Dimension

The third dimension is the so-called psychological factors, made up of individual psychological factors (intra-Individual skills category) and collective psychological factors (inter-individual skills category).

With regard to psychological factors, practically all subjects consider that this dimension is quite important to reach the elite and, above all, to stay in it. In this sense, many of the players refer to motivation as one of the key factors to fully develop a successful career in the elite (S1, S2, S3, S6, S7, S8, S9, S10, S11). When many of the subjects referred to motivational aspects that positively influenced their career, a concept closely related to the social context emerged—culture intra-individual skills. For several subjects, their greatest motivation was based on the Chinese concept of “family honor”—to be able to get as high as possible in the world of sports in order to bring honor, glory and recognition to their family (S1, S2, S6, S8, S10):

“My greatest motivation to become a professional was not only based on me, I wanted my parents to be proud of me. For me it was important to be able to bring honor to my family so that they feel the respect of all the people”

(S6).

Within this category, another factor on which many players agreed was the importance of mental strength (S1, S2, S3, S4, S6, S7, S8, S9, S10, S11). Some subjects alluded to its positive presence in order to overcome possible obstacles throughout their sports career, such as post-injury or recovery processes (S6, S10), sports uncertainty regarding referring to sports aspects of his career (S1, S2, S4, S8, S9), or to overcome the extremely hard and exhaustive Chinese training system (S3, S8, S11).

“In the transition from the youth team to the senior team, in 2008, I was chosen for the national team, but I did not become a top-ranking player, having many minutes of play until the last three or four years. At the beginning in the team I was very lost, it was a difficult time for me, where I was injured and could not find my place in the team, also things were not going as well as before personally. I came to wonder if I had the talent to reach the top, but thanks to my persistence during these difficult times I did not give up. I kept trying and managed to get through that time”

(S10).

Finally, regarding individual psychological factors, several subjects referred to the importance of learning ability—of learning to learn in order to develop as players (S1, S2, S4, S5, S7, S8, S9, S10).

To a lesser degree of importance but worth mentioning, several subjects gave importance to leadership, highlighting it as an attribute that made them assume responsibilities throughout their sports career, leading to better personal and basketball development (S1, S4, S8, S9). For almost all subjects, the factors group cohesion and companionship were remarkable as something to keep in mind—sometimes, some subjects name them separately, and others talk about them together. Reference is made to the importance of these in basketball, keeping in mind that basketball is a team sport and individual success is usually achieved from group victories. (S2, S3, S4, S5, S6, S7, S8, S9, S10, S11):

“If your team performs well, you as an individual can more easily show your talent and your aptitudes. The team helps the personal growth of each player, so group cohesion is very important. The teams are the ones that really win championships”

(S11).

#### 3.2.4. Sport Specific Factors Dimension

The fourth dimension was the so-called specific factors of sport, resulting from the grouping of the categories “tactical factors,” “technical factors,” “physical capacities,” and “anthropometric factors.” Within this group, the category “tactical factors” was by far the most frequently mentioned by the interviewed subjects, quantitatively registering a greater number of units of meaning.

In the results of this category, it can be seen how several subjects highlight decision making as one of the most important, or the most important, of their basketball tactical knowledge (S1, S2, S3, S4, S6, S7, S8, S9, S11). In addition, several players relate the decision making of the players with the training methodology typical of Chinese culture to train the tactic with which they were trained as players; some compared it with methodologies received from other countries or international coaches (S1, S2, S3, S7, S8, S10). Regarding this methodology, aspects related to feedback, learning through scouting, or training comparatively through open/closed systems are pointed out. In another section, several of the subjects referred to the best tactics provided by shared competitive experiences with more experienced colleagues and rivals. These experiences could result both nationally (playing with expert and international players in CBA) or through their specific participation in other international leagues or tournaments (participating in foreign leagues such as NCAA or participation with the national team in the Olympic Games, World Championship, or Asian Championship) (S1, S2, S3, S7, S8, S9, S10, S11):

“Playing with the national team has helped me tactically. The international competitions experience has been important I have felt more leader in my club, I felt like a better player… Tactical abilities are important, to be a good player, you have to react according to different situations, in each match, There are always unexpected occasions and you have to know how you react to all changes. For example, the tournament in Poland helped me. Foreign players are good in tactical aspects, I think they have more adaptability, however, the Chinese always play systems more rigidly. Sometimes, I have learned to be more adaptable to tactical situations thanks to playing many minutes with foreign teammates”

(S9).

Finally, regarding the tactical factors, it is worth noting that, for three of the subjects (S4, S7, S10), much of their tactical learning was the result of observing basketball in an extra way—that is, watch CBA, NBA, or even your own games in your spare time.

Regarding the technical factors category, different players highlight various technical aspects, although in particular, there is one aspect in which many agree—the importance of defensive technique—considering this aspect as something quite important once the elite has been reached (S1, S2, S3, S5, S8, S9, S10). In addition, two subjects pointed out relevant cultural and methodological differences when working on technical capabilities (not only defensive technique, but any technical aspect) (S2, S7).

“For example, every summer I went to train with coaches to the United States. They work differently, much importance is given to individual work, while in China, almost all work of any aspect is group work. I would say that in China a more generic work is done, and in the USA a truly individualized work. Therefore, whenever I can, I hire American private trainers out of season”

(S7).

Within this category, some subjects also refer to the importance of shooting technique. It was key in their development as players to have a prominent role in games, giving them a great scoring ability, which consequently meant having a great impact on the game and on the scoreboard (S1, S4, S6, S7, S11).

Moving on to the physical capabilities category, the subjects highlighted three aspects to be highlighted within the “physical capabilities” category. The first aspect was the relevance of strength as a physical ability, especially being mentioned mostly by those players who played forward, power forward or center positions. (S1, S2, S3, S5, S8, S11). The faculty in which to support its game or variable to train especially to be able to reach the elite was mentioned as an attribute. The second highlight was the importance of agility and endurance, these being highlighted at times individually and at others jointly by several subjects. Mainly, they praised the importance of one or both of these physical abilities—mainly those players who occupied base or shooting guard positions on the court (S4, S6, S7, S9, S10, S11). The third and final highlight of this block was the work of flexibility as an injury prevention measure. Only two players made mention of this section (S5, S8); in one of these two cases, it was a real advance in their career, being considered by the subject as a significant aspect to highlight in his life itinerary (S8).

The category with the lowest number of units of meaning coded in the present study was “anthropometric factors.” Regarding this, some subjects made a mention of height as a factor that favors the vast majority of cases to reach and compete in the elite but clarify that it is not something totally essential. (S1, S2, S4, S8, S11). Even in the case of an exceptional height (S8), he considered that, with a lower height, his game would have had less impact, but that it was not fundamental for the development of his sports talent.

## 4. Discussion

### 4.1. Social Context Dimension

As in many studies that investigated the life path of athletes, family was one of the most relevant aspects for the career development of basketball players in this study [13,26,38,39,58,59,60,61,62,63], showing this relationship a positive influence throughout the entire sporting career. In the present investigation, it can be seen how this positive influence exists, serving as the main support for the subjects both in sports and non-sports decisions (transfers, source of motivation, etc.). Furthermore, in most of the interviewees, there is an influence of parents or direct relatives on the sport of specialization of their children [62], since, in many of the cases of this research they had been professional athletes (basketball or some other professional sports discipline). It is important to consider, in order to put in context the great influence of the family on the life itinerary of the study sample, the concept of “family tradition” from a Chinese historical–cultural perspective. For many of the subjects, as some studies described on Chinese citizenship [64,65], family tradition is a broad concept, based on the idea of honoring and respecting the family in everything. Once this is considered as a basis, it can be understood that several of the interviewees tried to continue the family tradition, which was the cause of their involvement in basketball and one of their main motivations. According to the values described by Confucianism, which prevail in most families in China, respecting one’s elders, respecting authority, taking care of your family, honoring their memory, and honoring them with all ones actions should be the most important thing for a Chinese citizen [64,65,66]. This is not the only aspect of the traditional Chinese family to be taken into account, since the “One Child Policy” that prevailed until 2015 in China has various lines of influence on the development of sports talent. On the one hand, in this context there are no positive influences studied coming from older or younger siblings, such as those who speak of their role as role models, learning facilitators, or transmitters of skills, values and work ethic [14,26,67,68]. On the other hand, other studies that talk about the negative effect—jealousy between siblings—that can influence the development of talent; specifically, the special treatment or favoritism of parents toward a special child [69].

Regarding education, several authors have investigated the known dual career pathways in the literature, especially regarding their career transitions and their post-retirement phases [23,70,71,72]. In the present study, many of the players interviewed referred to the sporting benefits provided by having attended upper secondary education and university education. These benefits alluded both to an increase in concentration and learning capacity and an improvement in the tactical comprehension capacity of the basketball concepts transmitted by the coach, which added to those natural from the game’s own development. These results largely coincide with what Aquilina [73] expounded in her multi-sport study: that sports benefits such as better intellectual stimulation, better learning abilities, or the acquisition of better coping strategies facing injury recovering obtained in some athletes as a consequence of adequate formal education. Maintaining a balance between sports and academic life and the benefits that this balance brings to athletes has highlighted the benefits for social life and the development of strategies that help subjects manage their sports life [74]. In the case of the results obtained in our investigation, this lack of balance is latent, caused by the academic abandonment of the vast majority of Chinese basketball players. Along these lines, and in conjunction with these results, García-Naviera and Villanueva [75] talk about the cognitive benefits that the educational system provides for players, this effect being somewhat wasted on those athletes who drop out of academic studies early.

In another section, it can be seen how the results obtained on the importance of the city of birth coincide with numerous studies mentioned in the theoretical framework of this document, on the relevance of the size of the city of birth, and its relationship with the opportunities to achieve sports elite [6,76,77,78,79,80,81]. As in the example proposed in the United Kingdom, the results obtained in this study refer to the fact that “medium-sized” cities are more likely to produce elite athletes than anywhere else, with urban centers of between 30,000–1,000,000 residents being the most likely locations for the development of Olympic athletes [82]. Côté et al. [78], who, after studying the cases of basketball players, hockey players, baseball players, and golfers in the United States, determined that “average city” of between 50,000–100,000 inhabitants is the place that offers greater opportunities for the path to the sports elite. In the case of China, it is appropriate to make an important observation in this regard, since the population figures should be adapted to the size of the country in order to correctly categorize the name of “medium-sized city.” Several of the players interviewed spoke about the cities of Shenyang (Liaoning) and Tangshan (Hebei) referring to them as “medium cities,” these being, according to official data from the 2019 Chinese Census, cities with just over 8,200,000 and 7,100,000 inhabitants, respectively. Thus, considering the most important populations in China such as Beijing (21,500,000), Shanghai (24,000,000), or Guanghzou (19,000,000), it can be considered in the first instance that future research on birthplaces in the context of China would be interesting to assess this criterion.

Regarding idols, the results of this research corroborate what has been exposed by numerous studies that affirm that sports idols are a great influence and inspiration for young boys and girls who may consider dedicating their lives to sport as a consequence of great professional admiration. and personal by these [83,84]. Studies carried out specifically on Chinese adolescents concluded that, in China, the biggest national Olympic stars, foreign soccer players, and NBA players are the most common idols of the country’s youth [85].

### 4.2. Sport Context Dimension

The great influence that coaches have on the development of their trainees’ expertise has been the subject of several investigations, coinciding with the results obtained in this investigation, finding the positive influence that coaches exert on the development of the talent of athletes [59,86,87,88,89]. In addition, this influence is not limited only to sports aspects, but also encompasses personal aspects, coming to exercise a figure of mentor or “father,” who helps the player in many aspects of his life on and off the court. This circumstance was also found to be fundamental to reach the sports elite in other studies [9,13,40,88,90,91]. Another aspect highlighted by the interviewed Chinese players in relation to the coaches, is their ability to detect the talent of the players, being one of the first links in the talent detection system. This function on the part of the first trainers, was also highlighted by Christensen [92] who made reference to the “coach’s eye” and how they used their practical sense and visual experience, to recognize specific movement patterns among talented subjects.

Most of the players interviewed in this study highlighted the excessive number of hours spent training, as well as the poor preparation for training. In this line, it must be taken into account that the number of hours of training is not a synonym indicator of expertise, and that training without permanent concentration does not imply improvement in performance [93]. In addition, training focused only on the competition and professionalization of the athlete applied at an early age is associated with negative effects such as burnout, high risk of injury or the inability to develop transfer in their abilities [41,89,94,95,96,97,98,99,100]. Some research in sports such as basketball, skating, hockey or wrestling, justify that coaches should try to maximize the quality and efficiency of each session, instead of seeking to increase the volume of training hours [98,99]. In this sense, the Chinese socio-cultural prism should be kept in mind, which it describes as an adequate professional ethic, one that is based on long and long days [100]. However, as mentioned in the theoretical framework of this document, there are several studies that show that there are also sports success stories of athletes who accumulated much less than 10,000 h of deliberate practice [8,101,102,103,104,105]. Furthermore, they highlighted that in sports considered tactically as “open”, as is the case of basketball, the influence of deliberate practice shows a lesser influence than in closed modalities, where the environment is better known [103].

Within the sports context, something also highlighted by several subjects was their positive feeling after international experiences such as participation in the Olympic Games, stages with NBA teams, World Championships, or in Asia, which made them perceive a great improvement and growth as players. This similar effect was discovered in tennis players, concluding that there is an increase in the level of players when they play against rivals of a level equal to or higher than theirs, being important to originate sports opportunities where the best players can face each other so that the improvement curve grows as much as possible [12].

Regarding the agent’s role, as part of the industry agents can be an aspect to take into account in everything related to transfers between clubs [104], especially regarding transfers at the international level [105], therefore, in order to have more international experiences, having the services of an agent could be a factor to consider for Chinese players with sports potential who want to experience other leagues or tournaments in other countries.

Another result within the Sports Context refers to sports facilities—specifically, the increase in the creation of basketball courts in China since the “Yao Ming” effect in 2003 (the result of the popularization of basketball among citizens) [106]. It is considered important that the facilities of a specific sport required are appropriate in number and of good accessibility, so that there is a favorable system for developing a sport in a country or region, allowing it to be sufficiently nourished by athletes [107]. This phenomenon can affect the number of players who practice the sport and, therefore, the pyramid system of talent development, as for example occurs in the training of professional players in tennis and golf, where the prices and the accessibility of potential Players to practice the sport itself is considered a factor to take into account [108].

Lastly in this dimension, several of the interviewees highlighted the influence of the “luck factor” throughout their life itinerary. Similar results were obtained in their research with Spanish basketball players, regarding the luck of not suffering long-term injuries and luck of enjoying sports opportunities based on the injuries of other teammates that led to opportunities [109].

### 4.3. Psychological Factors Dimension

Among the intra-individual skills, one of the most repeated and highlighted comments by the interviewed players made reference to motivation as an engine in their sports career. This follows the line of studies that describe the same situation for non-elite players [110,111,112] elite junior players [113,114,115], elite players [57,116,117,118], and super elite players [31,38,69,119,120,121,122,123,124], where motivation was considered a key element to overcome obstacles throughout the athlete’s career, both sports and non-sports sports. These high levels of motivation are often closely related to other psychological traits beneficial for achieving sporting success, such as resilience or mental strength [31,112,113,123]. In the case of the subjects interviewed in the present study, much of the motivation came from the following three major sources of inspiration:

- The desire to attribute glory and honor to the family, to make their family proud, understood under the concept of “Chinese family tradition” [64,124].
- The desire to bring glory to the country, raising it to the highest as the term “Juguo Tizhi” encourages Chinese citizens to do [125].
- Love and passion for basketball. As a fuel for motivation, similar to that described by previous authors [126,127]. This concept would also be close to the attraction for basketball, or sport enjoyment that defines the Sports Commitment Model [128].

Mental strength was another aspect highlighted by the interviewed subjects, being considered as a requirement to face the different sporting challenges and to successfully overcome the tough Chinese training system and methodology. A review on the exact definition and all the nuances of the term “mental strength” was prepared by Crust [129], highlighting aspects related to the ability to face stressful situations, resilience, and the ability to bear greater physical loads (e.g., rowing) or psychological (e.g., in artistic gymnastics). Regarding mental strength, it was possible to see how the super-elite athletes compared to the elite, had overcome a critical negative event during their training stages (e.g., divorce from their parents, death of a relative), soon followed by a critical moment positive in sports [31]. Along these same lines, mental strength is closely linked to the PCEs or psychological characteristics of excellence [118], where he explains that the ability of athletes to handle stressful situations is key to their path to the top of the sports elite. The idea is even introduced in other articles [130,131], pointed out that “talent needs trauma”, understood this trauma as an adequate level of challenge in the tasks that act as facilitator of learning, use and refinement of psycho-behavioral skills, which will ultimately favor the development of the athlete.

To a lesser extent, the learning capacity of the players was also highlighted, especially with regard to the ability to adapt and assimilate basketball concepts. This coincides with what was expressed by Issurin [132], when he spoke that the assimilation and learning capacity that each athlete presents can be differentiating, since deliberate practice is the source of experience, but it will also have to be considered that this practice is assimilated in its fullness [35,59,132,133].

Among the collective psychological factors, one of the highlighted results refers to how the fact of assuming leadership within a team meant an increase in responsibilities on and off the court, and consequently, this was implicitly associated with personal growth and sporty at various stages of their careers. This coincides perfectly with the longitudinal study by Mead, Gilson and Henning [134] where, after evaluating the changes experienced by the captains of various teams during an entire season, they concluded that there was a great increase in the captains of their self-confidence, ability to motivate peers, ability to be heard, and personal growth and maturity [134].

Another of the results highlighted within collective psychological factors, made reference to the importance of group cohesion and its relationship with collective success. This is aligned with studies that conclude that good group cohesion can help the development of the athlete’s career in aspects such as obtaining better group results [135,136,137], emotional stability in the face of life changes [138,139,140,141], facilitate learning about values, common goals and roles in the team [142,143,144], and increase group motivation by reducing the burnout percentage [137,142,143].

### 4.4. Sport Specific Factors Dimension

Within the Tactical Factors dimension, the interviewed subjects made references to the great importance of decision-making on the game and the sporting impact that good decision-making has on the sports career of any basketball player, highlighting this factor well above of any other result within this category. Many studies share this idea, highlighting the importance of the tactical component in team sports [4,10,103,145,146,147,148,149,150] and its close relationship with a successful performance in basketball [39,61,92,121].

Specifically, the interviewees in the present investigation explained how good decision-making positively impacts the development of the player’s talent. The influence of sports activities “unstructured” by an adult (i.e., deliberate play), as a developer of the capacity of the decision-making subject, as well as the presence of a multi-sport practice in training stages in those players with better decision making [98,151]. Later, other studies have also studied the benefits of deliberate play with respect to tactical creativity and tactical intelligence, observing better performance in those subjects who enjoyed this unstructured training [149,150]. Within this unstructured practice, it was observed how invasion activities were associated with a greater improvement in tactical decision-making [151]. In the case of the sample of the present study, none of the subjects had a significant amount of deliberate play or enjoyed multidisciplinary sports training.

Based on the low frequency of units of meaning presented by the categories Technical Capacities, Physical Capacities and Anthropometric Factors and the similarities in the characteristics of their results, these will be discussed together. In general, this group of categories showed two common characteristics in all the categories that comprise it:

- The results obtained could be classified as properties necessary to reach the elite, but not sufficient for it (e.g., adequate height, good shooting technique, etc.).
- The results obtained were widely contrasted, accepted and consolidated in the scientific literature, not assuming any kind of contribution to the field of knowledge in which this article is framed (e.g., the required strength training, or the positive influence of a good endurance within the Physical Capacities).

The only notable aspect at the scientific level was the differentiation by the interviewed subjects of the way of working with Technical Capacities and Physical Capacities in China compared to the international approach. The players highlighted the more individualized character of the international methodology, in contrast to the collective character of the Chinese methodology. In this regard, it should be noted that in China there is an increasing presence of specialized physical capacity training programs by positions [152,153], these being a methodology not traditionally used, but with increasing popularity in the country both in university basketball [152] and professional basketball [153].

### 4.5. Cultural Factors

As described in the results of this document, in several of the dimensions, aspects related to culture arose inductively. From a holistic point of view, a large part of the units of meaning were intrinsically and constantly impregnated with cultural aspects, above all, in the way in which the interviewed subjects understood certain specific personal, social and sporting aspects as “normal” (e.g., the coaches’ way of acting, their own way of acting based on what their family and country accept socially, etc.). Based on these arguments, throughout the sports results, actions, training methodologies, family influences, etc. have been justified.

Samples of the cultural weight in the Chinese talent development model can be seen from its base, since it is a model where a common emphasis is placed on early specialization, of a single sport, very focused on the identification of talent and not so much on the development of same, where the family and cultural tradition condition different characteristics and factors throughout the athletes’ career (e.g., type of chosen sport, motivations to reach the elite, high nonexistence of fraternal influence, early abandonment of the academic field, methodology of traditionally analytical training applied by many Chinese coaches, etc.). From a different angle, national sport culture also matters, taking as an example in the case of China the “Yao Ming effect”, which helped to increase the popularity of basketball in the country, mainly supported by the increase in the number of players, the number of facilities available and the number of specialized schools [106]. Taking into account all the latest works described in this section, and based on the results obtained, cultural factors could be considered as factors that permeate and are present in the different factors that condition talent development.

### 4.6. Study Limitations and Future Research Lines

This research has limitations that investigators have tried to reduce to a minimum in order to avoid errors in the interpretation of the conclusions. (a) the available time of the interviews. Sometimes, the lack of time of the players caused some of the interviews to be shorter than desirable. Although in all of them it was possible to deepen the foreseen topics, in some cases, the ideal interview time would have been longer than the one that occurred. (b) This research has focused on expert basketball players in China. Therefore, when extrapolating the conclusions to other types of talent development societies and systems, it is possible that the conclusions are limited to the characteristics of this specific country. However, the opportunity to unravel some information of this enigmatic country has been very interesting on a scientific level.

Future lines of research could analyze the effects of cultural factors on other top basketball countries which remain unexplored (e.g., Argentina or Australia). Also, to replicate this study on female players or combining qualitative and quantitative methodology could be interesting.

## 5. Conclusions

After the discussion of the results, the following research conclusions are presented. Responding to the objectives of the investigation, we can identify the following factors of great importance in the development of basketball talent in China.

The cultural factor and its influence on the different dimensions should be considered as a determinant in the development of talent in basketball in China and very probably in any other research carried out in another country. This factor should be considered as a filter to be applied to understand the training process of an athlete. An example of this can be seen in the social context, where “family tradition”—deeply rooted and valued in Chinese culture—is an element with high influence on the players, both when choosing basketball as a dedication and as a motivating element to overcome difficulties throughout their career. The concept of family tradition, with all the cultural nuances associated in China, is the main motivation to reach the elite for many of the subjects.

Finally, regarding the social context, Chinese elite players value negatively the abandonment of academic studies at young ages in favor of basketball exclusive dedication.

The participants describe their coaches as an important figure in their development, although they also reflect a low assessment of their methodological knowledge, mainly based on the long and monotonous training sessions. The classical methodology commonly used in China, in comparison to other nations, requires updates on coaches’ feedback and training individualization, having as a result a less effective development due to the absence of these elements. Training during the initial stages of Chinese athletes is characterized by early specialization, as opposed to an early diversification model of different sports disciplines, which is not common in China.

Chinese elite players consider decision making an important factor, for which playing international competitions and competing with/against foreign players enrich their tactical ability, thus positively influencing their sports development as players. Mental strength was an essential element to overcome China’s hard training methodology and to face all kinds of difficulties that may arise throughout the race. Further investigations are required to confirm the findings of the present study.

From these findings, different practical applications could emerge, such as the creation of a department of sports psychology in the Chinese basketball federation or the different Chinese CBA clubs, which supports athletes throughout their development toward the elite and that teach athletes coping strategies to face difficult times on and off the court. Another practical application could be the creation of a modern, renewed, and homogeneous professional development program in all provinces for coaches, mainly focused on methodology and feedback techniques similar to those used in other countries of international basketball prestige.

## Figures and Tables

**Table 1:** Interview structure.

Dimension	Content Examples
Social Context	Family influence, academic studies, couple influence.
Sport Context	Coaches influence, sports facilities, training structure.
Intra-Individual Factors	Motivation, leadership.
Inter-Individual Factors	Companionship, roles in the team.
Technical Capacity	Dribbling, shooting technique.
Tactical Capacity	Movement with and without the ball, decision making.
Physical Capacities	Strength, speed, agility, endurance.
Anthropometry	Height, weight, span.
